# Integration of ancient DNA with transdisciplinary dataset finds strong support for Inca resettlement in the south Peruvian coast

**DOI:** 10.1073/pnas.2005965117

**Published:** 2020-07-13

**Authors:** Jacob L. Bongers, Nathan Nakatsuka, Colleen O’Shea, Thomas K. Harper, Henry Tantaleán, Charles Stanish, Lars Fehren-Schmitz

**Affiliations:** ^a^Sainsbury Research Unit, University of East Anglia, NR4 7TJ Norwich, United Kingdom;; ^b^Cotsen Institute of Archaeology, University of California, Los Angeles, CA 90024;; ^c^Department of Genetics, Harvard Medical School, Boston, MA 02115;; ^d^Conservation Department, Fine Arts Museums of San Francisco, San Francisco, CA 94118;; ^e^Department of Anthropology, The Pennsylvania State University, University Park, PA 16802;; ^f^Escuela Profesional de Arqueología, Universidad Nacional Mayor de San Marcos, Lima 15081, Peru;; ^g^Institute for the Advanced Study of Culture and the Environment, University of South Florida, Tampa, FL 33620-8100;; ^h^University of California, Santa Cruz (UCSC) Paleogenomics, Department of Anthropology, University of California, Santa Cruz, CA 95064;; ^i^UCSC Genomics Institute, University of California, Santa Cruz, CA 95064

**Keywords:** ancient DNA, mobility, Andes, transdisciplinary approach, Inca

## Abstract

Ancient DNA (aDNA) analysis provides a powerful means of investigating human migration, social organization, and a plethora of other crucial questions about humanity’s past. Recently, specialists have suggested that the ideal research design involving aDNA would include multiple independent lines of evidence. In this paper, we adopt a transdisciplinary approach integrating aDNA with archaeological, biogeochemical, and historical data to investigate six individuals found in two cemeteries that date to the Late Horizon (1400 to 1532 CE) and Colonial (1532 to 1825 CE) periods in the Chincha Valley of southern Peru. Genomic analyses indicate that these individuals are genetically most similar to ancient and present-day populations from the north Peruvian coast located several hundred kilometers away. These genomic data are consistent with 16th century written records as well as ceramic, textile, and isotopic data. These results provide some of the strongest evidence yet of state-sponsored resettlement in the pre-Colonial Andes. This study highlights the power of transdisciplinary research designs when using aDNA data and sets a methodological standard for investigating ancient mobility in complex societies.

## Significance

Genomic, archaeological, historical, and biogeochemical data are integrated to examine six individuals from two cemeteries in the Chincha Valley of southern Peru. Results demonstrate consistency among these independent datasets in support of a model of north Peruvian coast peoples moving to the Chincha Valley during the Late Horizon (1400 to 1532 CE). Our transdisciplinary work provides strong support for Inca resettlement, a state policy that reshaped the Andean sociopolitical landscape yet represents one of the most notoriously difficult phenomena to identify in the archaeological record. This research offers an ideal case study that sets a methodological standard for investigating ancient mobility in complex societies by synthesizing aDNA with multiple independent lines of evidence.

Analysis of ancient DNA (aDNA) is a powerful method for studying the past (1). Developments in next generation sequencing technologies and reliable authentication protocols have made genome-wide analyses of large sample sizes a reality (2). Genome-wide data are critical for studying ancient mobility because they enable researchers to infer ancestry of groups and individuals in different regions with high precision. This can point to changes in ancestry over time in the same region, or even groups of different ancestry coexisting in the same area. Both patterns provide evidence of migration into the region, including recent unadmixed immigrants or mixture of the new migrants with previously existing groups.

Conclusions drawn from studies of aDNA, however, have not been immune to criticism. Some argue that these research designs tend to favor continental- and global-scale genetic analyses (3), resulting in “grand narratives over time and space” (4) that do not sufficiently model movement at more local scales. Others note that while authors of genetic papers effectively identify movements of people, they rarely provide enough context or theory to explain how and why such movements occur (3–5). Issues of scale, context, and sample size limit understandings of mobility because they result in problematic assumptions of archaeological cultures as people. Genetic studies also tend to treat migration as merely an explanatory “black box” (6). Critics of genetic studies have made it clear that no single type of data, including aDNA, can identify and explain mobility in isolation. These complex research questions demand a multi- and transdisciplinary approach.

**Fig. 1.**
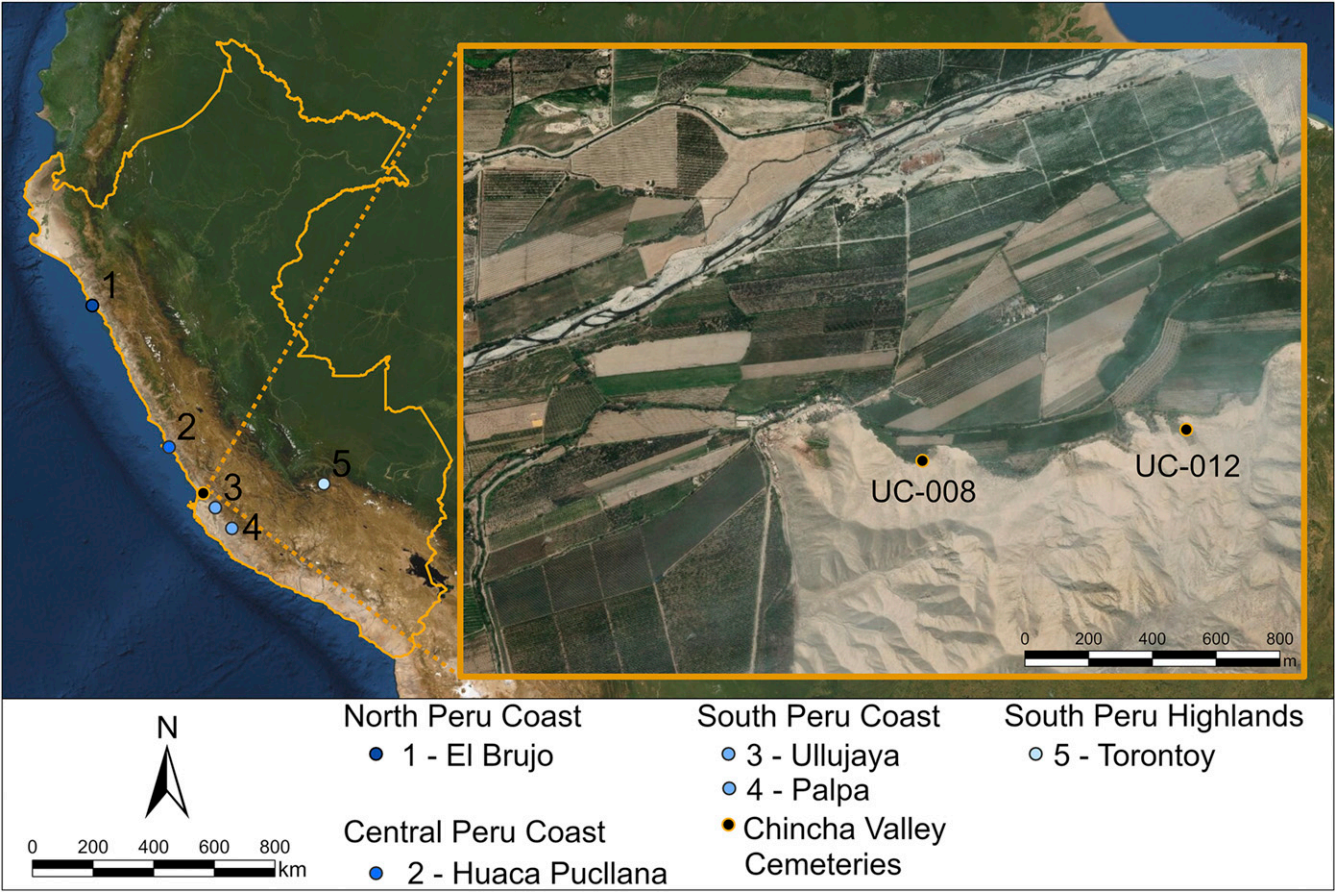
Map showing the locations of the Chincha Valley, the cemeteries under investigation, and other archaeological sites in the Andes that yielded aDNA data cited in this study.

It is crucial to integrate distinct types of data and evaluate the extent to which each type tests anthropological models of movement at various times and population scales. Here we employ such a transdisciplinary approach that synthesizes multiple, independent lines of evidence at a local scale to identify, contextualize, and explain mobility in complex societies more effectively. This transdisciplinary approach has been employed in few genetic studies (7, 8), and we demonstrate it with a case study from the Chincha Valley, located on the south Peruvian coast (Fig. 1). The Chincha Valley is well suited for this investigation because it was reportedly targeted as a destination for Inca-influenced movement of nonlocal peoples in the 15th century, and its arid conditions permit the preservation of critical material markers of identity (e.g., decorated artifacts) as well as human remains that are conducive to aDNA analysis. The Inca were among the few societies that uprooted men, women, and children and moved them away from their homeland. This Inca state policy of forced resettlement transformed the Andean sociopolitical landscape, yet remains difficult to identify in the archaeological record.

## Ethics Statement

In this study we integrate archaeological, historical, isotopic, and genomic data as well as the perspectives of the indigenous peoples of Peru. Our work was conducted in accordance with the wishes of these communities. Groups who are geographically proximate to the graves in the middle Chincha Valley expressed interest in learning more about the past peoples who lived in the area because all documented mortuary contexts have been visibly disturbed. This study is the result of a broad research program that has fostered international collaboration in archaeological endeavors among university students from the United States and Peru and local community members from the Chincha Valley (2012 to present). This study is fully authorized with permits from the Peruvian Ministry of Culture. Representatives of indigenous peoples were part of this supervision process. We complied with all legal and ethical norms for the study of aDNA. Our research demonstrates that disturbed archaeological sites can provide scientifically useable data to archaeologists and indigenous groups. We will continue to work with local leaders and the Chincha municipal museum to share our research findings with indigenous communities.

## A Case Study in Transdisciplinary Research

Mobility is widely recognized as a crucial component of human behavior (9), and it is a complex, variable, and potentially structured process (10). Whether fleeing from climate change (11) or military threats, resettling in a distant locale under state influence (12), or traveling to ecologically distinct areas to exploit resources (13), people in the Andes and elsewhere move—voluntarily and otherwise—for a variety of reasons. Understanding why people move is crucial for understanding paleodemography in particular, and human history in general. Researchers have examined mobility using tools from archaeology, bioarchaeology, biogeochemistry, linguistics, and, most recently, genetics.

A complex polity known as the Chincha Kingdom controlled the Chincha Valley in the Late Intermediate Period (LIP; 1000 to 1400 CE) along the south Peruvian coast. Sixteenth century documents describe the Chincha Kingdom as a wealthy society comprising a network of interdependent communities of specialists including farmers, fisherfolk, and merchants (14). During the Late Horizon (LH; 1400 to 1532 CE), the Inca consolidated their power in the Chincha Valley, resulting in an expansion of trade networks, installment of a “dual rule” political system headed by Inca and Chincha lords (15), and forced movement of nonlocal settlers (*mitmaqkuna* or *mitimaes*) into the area (16).

Recent archaeological research in the middle Chincha Valley (17) reveals a dense distribution of more than 500 graves that cluster in 44 mortuary sites. There are two distinct grave types: 1) subterranean cists and 2) above-ground and subterranean graves resembling mausolea (*chullpas*). For this paper, two subterranean *chullpas* made of fieldstone were selected for aDNA, radiogenic strontium isotope, and textile analyses: UC-008 Tomb 1 from the UC-008 cemetery (0.48 ha) and UC-012 Tomb 33 from the UC-012 cemetery (also known as Pampa de los Gentiles) (1.57 ha). These cemeteries (*SI Appendix*, Fig. S1) are ∼950 m apart (Fig. 1). UC-008 Tomb 1 (3.09 × 2.35 m) contains at least 117 people as well as textiles, ceramics, and fragments of maize and gourds (17, 18). UC-012 Tomb 33 (2.15 × 2.10 m) has an unknown number of multiple individuals deposited alongside textiles, maize, and gourds. Four previously published ^14^C AMS (accelerator mass spectrometry) dates (18) from UC-008 Tomb 1 indicate grave use in the Late Horizon and Colonial period (1532 to 1825 CE). Since no Colonial-era artifacts have been recovered in the middle Chincha Valley, these dates suggest that the individuals in this tomb are from the Late Horizon.

We first review several lines of evidence from the Chincha Valley that suggest mobility during the Late Horizon. These include ceramic, textile, and strontium isotope data, and Colonial-era records claiming that the Inca moved nonlocals across the south coast, including the Chincha Valley, as part of their imperial policy. Limitations of each line of evidence are highlighted to reinforce the point that no single type of dataset alone can identify mobility and explain how and why people moved. Subsequently, genome-wide data from six individuals deposited in UC-008 Tomb 1 and UC-012 Tomb 33, accompanied by direct ^14^C AMS dates for four of those individuals, are presented.

## Evidence of Migration into the Chincha Valley

### Ceramic Data.

**Fig. 2.**
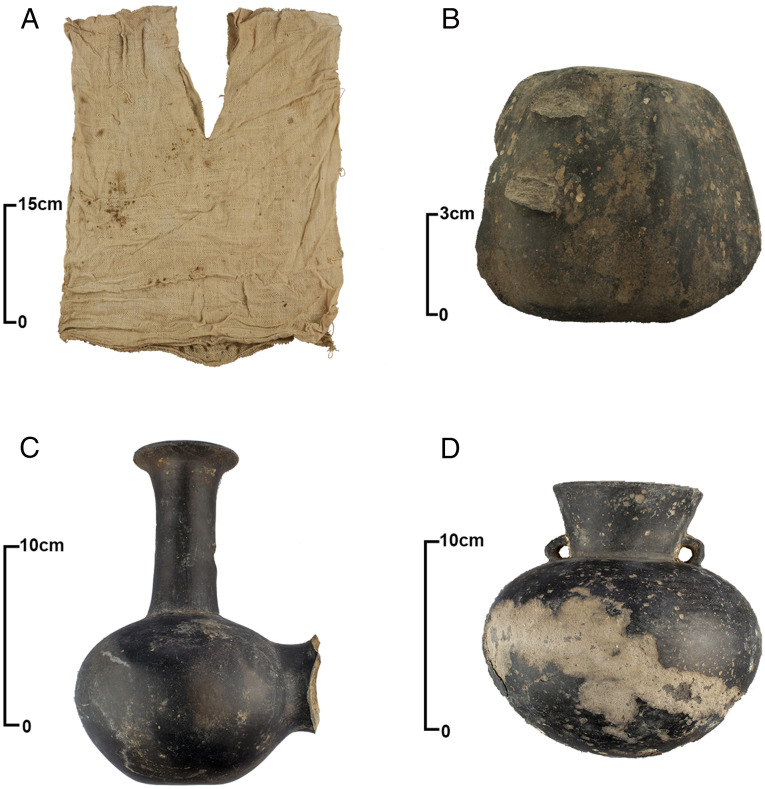
Objects from the middle Chincha Valley that appear to be Late Horizon in date and north coast in style or technique: (*A*) tunic (18.50 × 14.57 inches) from UC-008 Tomb 1 demonstrating a plain weave structure with paired warps and single wefts; (*B*) a ceramic fragment from UC-008 Tomb 1 that may have been part of a blackware, Chimú-Inca aríbalo (23); (*C*) a double-bodied, blackware bridge vessel from an adobe *chullpa* in the UC-044 cemetery; and (*D*) a blackware vessel from the UC-035 cemetery. Modified from ref. 18.

Material objects, such as ceramics, may not have reflected the fluidity of group identities because they can take on varying roles in social interaction through time and across different contexts (19). For example, in cases of trade, ideas or objects can spread without migration of people, but once pottery is traded, ceramics too can be copied and emulated with local clays. Properly contextualized, however, they remain an important indicator of potential mobility in the Andes (20). Previous archaeological research finds central- and north coast-style ceramics throughout the Chincha Valley. In the early 20th century, Max Uhle excavated six mortuary sites near the site of Huaca La Centinela in the lower Chincha Valley. An assemblage of 55 ceramic vessels recovered from graves dating to the Late Horizon was analyzed by Menzel (21). A variety of nonlocal ceramic styles were identified, including Pachacamac-Inca, Chimú, and “central to north coast” styles (21). Nine pottery bottles built in the shape of tubers and fruits are attributed to the central to north coast style (21). Vessels of this type were recovered from the site of Lo Demás, located in the lower Chincha Valley and excavated by Sandweiss (22). Menzel (21) also identified two vessels that represent the Chimú style: a stirrup spout bottle and an animal-headed bottle. North coast-style ceramics (23) have also been noted in recent archaeological research, including a survey of mortuary contexts in the middle Chincha Valley (17) (Fig. 2).

### Textile Data.

As another type of material object, textiles carry the same limitations as ceramic vessels, yet in the Andes, they are broadly viewed as one of the most important indicators of group identity (24). This is because ethnographic and archaeological research suggests that Andean weavers, both past and present, learned and transmitted technical knowledge in group environments, thereby establishing preferences in textile production practices that distinguished communities, cultures, and group identities (18, 25, 26). The Inca required *mitmaqkuna* to retain their traditional textile styles (27). For these reasons, textiles are considered as a “clear archaeological signature” (12) of *mitmaqkuna*, but they are not always preserved in the material record.

An abundance of Chimú-style textiles has been found across the study area. A salient feature of this north coast style is a plain weave structure with paired warps and single wefts (28). O’Neale et al.’s (29) analysis of textiles recovered from Uhle’s excavations discovered that most textiles in the assemblage (62 out of 112) have paired warps and single wefts. In her examination of 13 textiles from five graves excavated by Uhle, Garaventa (30) finds that the most common weave structure is plain weave with paired warps. A study of 127 loose threads and 42 fragments of cloth recovered from Lo Demás shows that not only are all textiles woven in the plain weave structure, but that most of these textiles feature paired warps and single wefts (22). Most recently, 141 textiles from UC-008 Tomb 1 were analyzed (18). These data demonstrate that 64% (90 out of 141) display the plain weave structure with paired warps (Fig. 2). We cannot rule out that the indigenous peoples of the Chincha Valley may have been influenced by north coast textile traditions or acquired these textiles through trade. These scenarios are unlikely, however, considering the difficulty in emulating weaving techniques from outside local areas where they are learned and ingrained as motor skills (31).

### Strontium Isotope Data.

Measurement of ^87^Sr/^86^Sr relative abundance ratios has been used to infer mobility in the Andes (32). While this approach can identify highland-coast movement, it currently cannot reveal movement among many coastal regions where geological signatures are homogeneous (33, 34). For example, strontium isotope ratios were measured in 42 tooth enamel samples from seven individuals found in UC-012 Tomb 33 (35). The ^87^Sr/^86^Sr relative abundance ratios range from 0.70618 to 0.70732 with a mean of 0.70706 ± 0.00032 (1σ, *n* = 42). In general, these data overlap with the ranges of ^87^Sr/^86^Sr values of archaeological soil samples from the Huarmey Valley of the north coast (0.70595 to 0.70792) (33), modern soil samples from the Lurín (0.70519 to 0.70805) and Rimac (0.70676 to 0.70712) Valleys of the central coast (36), and modern soil samples from the Pisco (0.70704 to 0.70801) and Ica (0.70711 to 0.70761) Valleys of the south coast (37). Thus, based on strontium isotope data alone, these individuals likely derive from a coastal Andean region, but their more precise geographic origins are unclear.

### Written Sources.

Despite being biased, the Colonial-era written sources of the Andes provide significant insights into mobility patterns, especially during the Late Horizon. These documents describe the Inca policy of forced resettlement. The Inca selected a significant number of a given population from each province and moved these *mitmaqkuna* to new destinations. These places varied in distance, but the state generally sought to put people in ecological zones similar to their home (12, 27). Prime agricultural land was given to *mitmaqkuna* (12, 27). Resettlement was primarily done to mitigate threats to Inca authority and support the state economy (13).

Cieza de León (16), an important soldier-chronicler of Peruvian history, indicates that *mitmaqkuna* were present in the Chincha Valley. The geographic origins of these *mitmaqkuna* are unclear, but considering other Colonial-era documents, it is likely that they came from the north coast. This is because after conquering the Chimú, quelling a subsequent uprising, and dismantling their political structure, the Inca reportedly resettled north coast groups previously affiliated with the Chimú (known as “Moche”) across the south coast. These included goldsmiths and metalworkers, fisherfolk, and canal and water management specialists (38). Moche *mitmaqkuna* were moved to the Acari Valley (39) and the Cañete Valley (40, 41). Two canal intakes in the Acari Valley are named Mochica Alta and Mochica Baja, and some canals are called Mochica in the Ica Valley. Since Mochica was the language spoken in the Moche region, these canal names suggest that north coast groups may have been placed on the south coast to construct and/or improve irrigation canals (38). The arrival of nonlocal groups may explain the increased population, irrigation, and agricultural production levels attested to during the LIP and Late Horizon in the Chincha Valley (42).

These lines of evidence raise questions concerning mobility: Were nonlocals present in the Chincha Valley before the Colonial period? If so, where did they come from, how did they get there, and why did they move? Is there any evidence that the Inca resettled nonlocals in Chincha? Integration of these lines of evidence with new, genome-wide data can address these questions with greater confidence.

## Genomic and Radiocarbon Results

We performed whole-genome sequencing to ∼0.71× to 1.65× coverage of six individuals who were deposited in two *chullpas* from two distinct cemeteries in the middle Chincha Valley. Two individuals are from UC-008 Tomb 1 and four are from UC-012 Tomb 33 (each of the four individuals from UC-012 were previously sampled for strontium isotope analysis). Our genome-wide analysis (below) identified the ancestries of these individuals with a high degree of precision, allowing for confident inferences to be made into their potential mobility patterns. General sequencing statistics can be found in Dataset S1. We obtained direct ^14^C AMS dates from the skeletal material of two UC-008 Tomb 1 individuals and two UC-012 Tomb 33 individuals. After correcting the dates for marine reservoir effect (*Methods*), the 95% confidence intervals of the dates for all individuals fell within the 1415 to 1805 CE range, with mean probabilities occupying the range of 1505 to 1645 CE (Dataset S1). These dates are consistent with the Late Horizon. The DNA of the individuals exhibited cytosine-to-thymine substitution rates at the ends of the sequenced fragments over 3% characteristic for authentic ancient DNA (43), and low point estimates of contamination in mitochondrial DNA (mtDNA) ranging from 1 to 2% (44). For three male individuals, point estimates of X-chromosome contamination also were determined to be very low, ranging from 0.6 to 2% (45). The genomes were merged with a dataset sequenced on the 1,240k single nucleotide polymorphism (SNP) array (46–48)_,_ consisting of genomic data from pre-Colonial period and modern Native American populations in South America, as well as other global populations (49–53). To increase our geospatial resolution and group diversity in the central Andes, we also performed additional analyses after merging our data with a larger set genotyped with the Human Origins array (HO, ∼550k SNPs), curating data of several Peruvian individuals throughout the region (54).

**Fig. 3.**
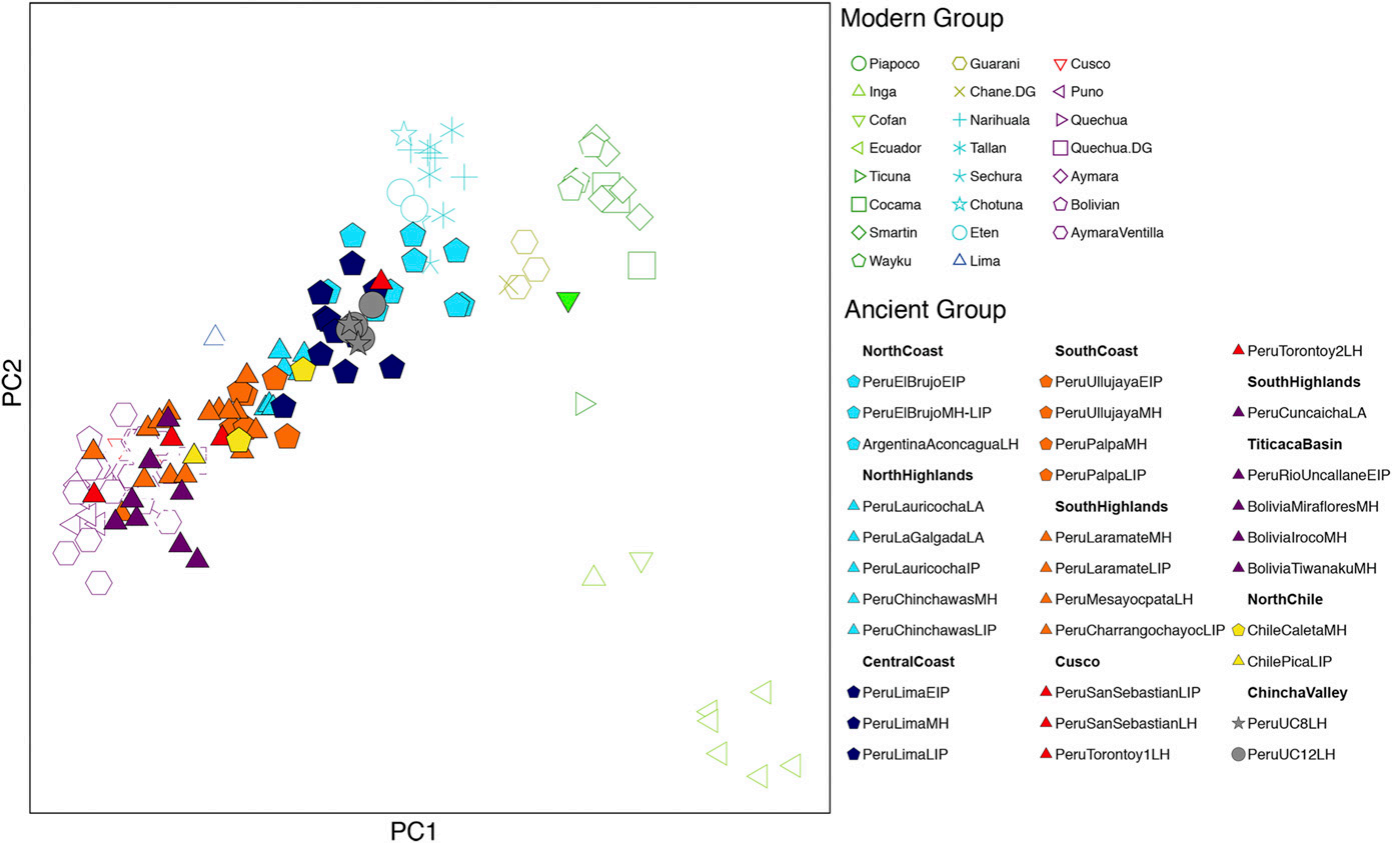
PCA of ancient individuals projected onto modern indigenous South Americans using the ∼550k SNPs overlapping with the Human Origins set. Modern individuals are unfilled; ancient individuals are filled. Color code is based on the geographic origin of the individuals (see legend).

We first determined that none of the six individuals are first- or second-degree relatives of each other using READ (55). Using the HO merged autosomal data, we then performed a qualitative assessment of the population structure on a per-individual basis using unsupervised ADMIXTURE analysis (*SI Appendix*, Fig. S2) and principal components analysis (PCA) (Fig. 3). The PCA replicates the observations made by Nakatsuka et al. (53), confirming population structure in the central Andes, roughly following a north–south cline, for both coastal and highland populations. The tested individuals from the Chincha Valley (Fig. 3, gray stars and circles) fall within the genetic variation of ancient and modern individuals from the north and central coast of Peru, and they are distant from the LIP and earlier individuals from the Ica and Palpa Valleys of the south Peruvian coast. The cluster analyses with ADMIXTURE confirm the observed structure, with the tested individuals showing ancestry compositions that are similar to Middle Horizon (MH) and LIP north coast and central coast individuals when considering K = 1 to K = 8, while the south coast individuals are either modeled as a mixture of coastal ancestry and highland ancestry (K = 4 to K = 6) or as exhibiting *SouthPeruHighlands* ancestry (K = 7 to K = 10; *SI Appendix*, Fig. S2).

The tested individuals from Chincha shared similar genetic ancestry, so we first assessed whether the individuals in each site (UC-008 or UC-012) were genetically homogenous by performing statistics of the form *f*_*4*_(*Mbuti, Test; UC12_Ind1, UC12_Ind2*) or *f*_*4*_(*Mbuti, Test; UC8_Ind1, UC8_Ind2*), where *Test* were all groups outside of the Chincha Valley or the other site, and taking all possible combinations of individuals within each site. None of the statistics were significant (Dataset S2*A*), showing that there is genetic homogeneity within each site (no *Test* groups share significantly more alleles with one of the individuals relative to the other). In addition, we used qpWave (56), which tests how many distinct ancestry sources are in a set of populations (“left” populations) relative to another set of populations (“right” or “outgroup” populations). The individuals within each site were consistent with one wave of ancestry when multiple Andean groups were used as outgroups (*P* > 0.01 for rank 0, Dataset S2*B*). These data indicate that the individuals in each site are homogeneous in ancestry, so we grouped them within each site.

**Fig. 4.**
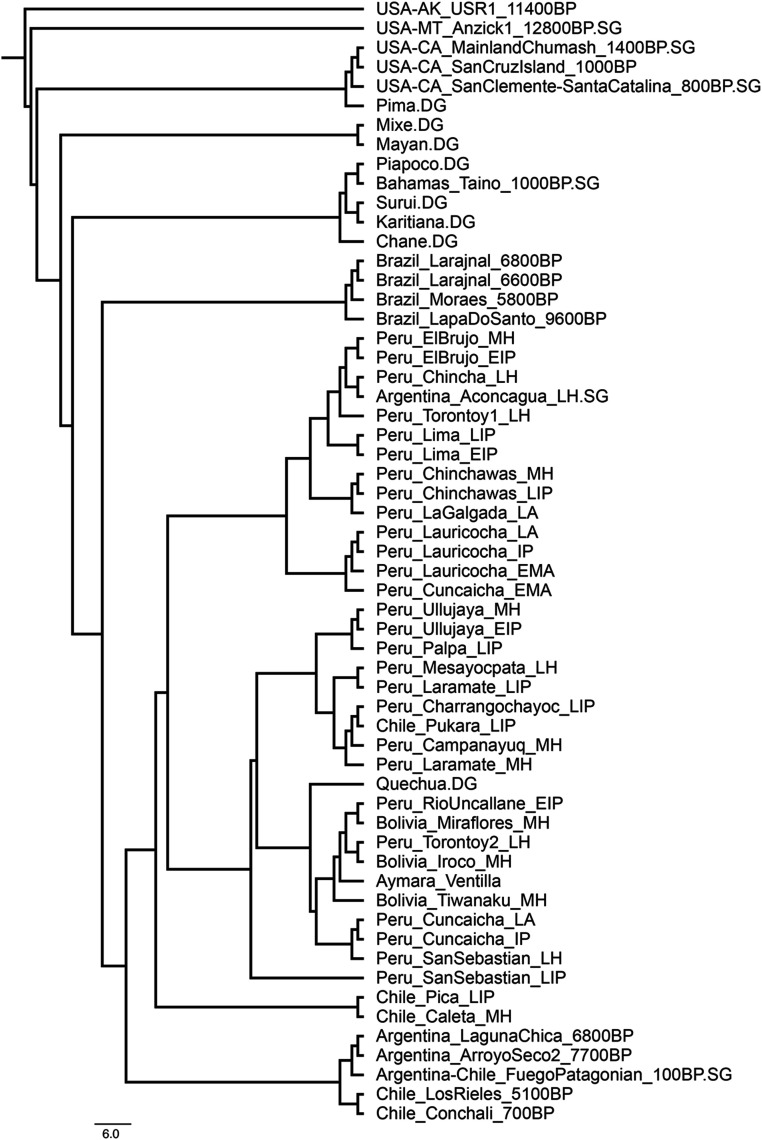
Neighbor-joining tree based on inverted outgroup-f_3_ statistics (1/*f*_*3*_[Mbuti; Group1, Group2]) using the 1,240k SNP set. The *USA_Ancient_Beringian.SG* individual (*USA-AK_USR1_Beringian_1140BP.SG*) was used as an outgroup for the tree. Only individuals with >25,000 SNPs are included.

**Fig. 5.**
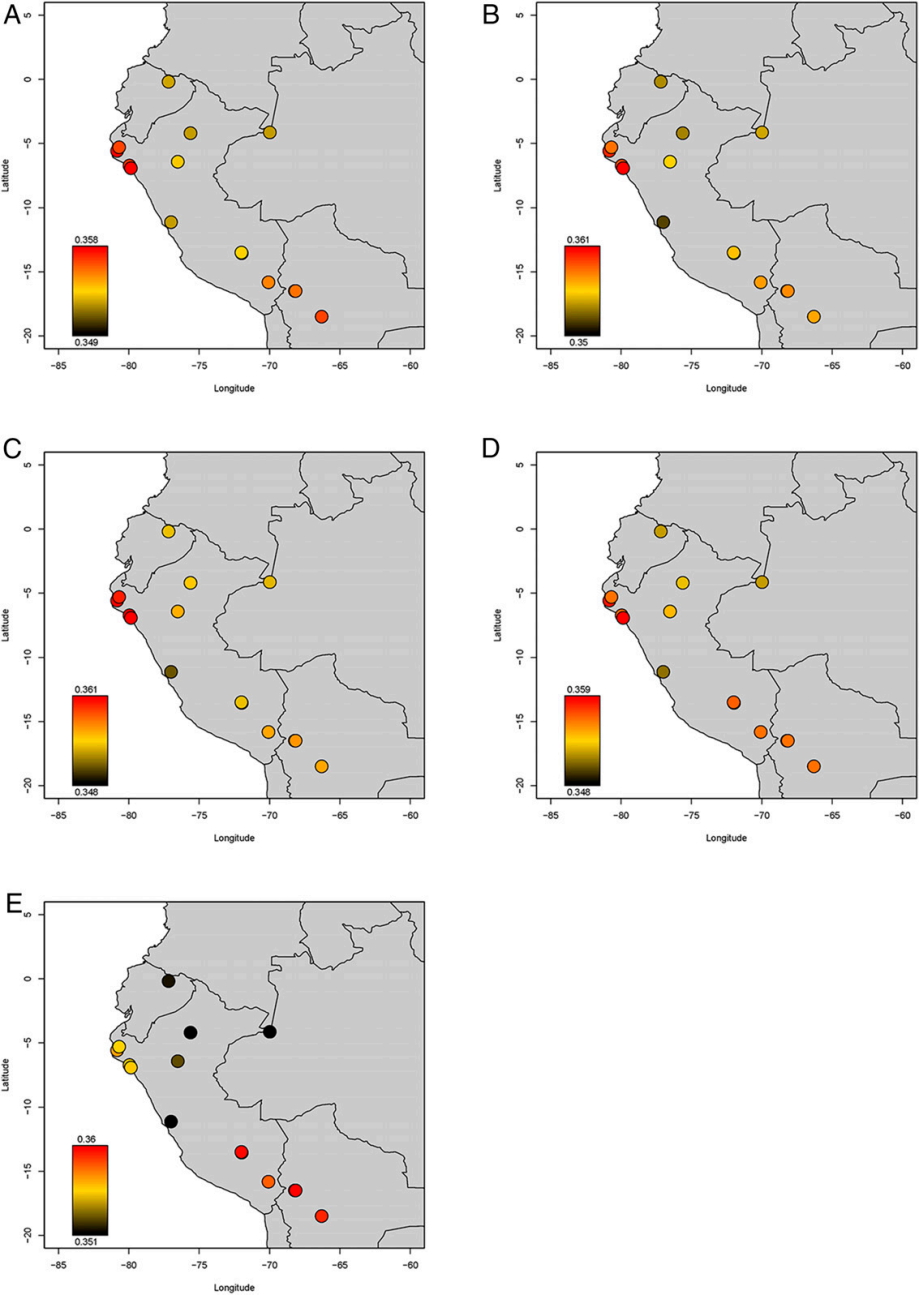
Heatmap of outgroup-*f*_*3*_ statistics. Color coding is based on statistics of the form *f*_*3*_(*Mbuti; Ancient, Modern*), where the ancient groups are (*A*) Peru_Chincha_UC8_LH, (*B*) Peru_Chincha_UC12_LH, (*C*) NorthPeruCoast, (*D*) CentralPeruCoast, and (*E*) SouthPeruCoast. Modern individuals are from Barbieri et al. (54).

Using the full 1,240k dataset we then generated a neighbor-joining tree of the matrix of “outgroup-*f*_*3*_” statistics of the form *f*_*3*_(*Mbuti; Pop1, Pop2*), which measures shared genetic drift between population pairs (Fig. 4). As with the PCA and ADMIXTURE, the Chincha sites cluster with or next to modern and ancient north and central coast groups (Fig. 3). In contrast, Peruvian south coast individuals from the Ica and Palpa region group branch with Peruvian south highlands individuals (Fig. 3) or close to them (Fig. 3). In addition, we computed statistics of the form *f*_*3*_(*Mbuti; Ancient Andean, Present-Day SouthAmerican*) (54) for UC-008 Tomb 1, UC-012 Tomb 33, and the three coastal groups, and visualized them as heatmaps in Fig. 5. Like ancient individuals from the north and central coasts, both Chincha groups show increased affinity with present-day indigenous groups from the north coast, while the ancient individuals from the south Peruvian coast show increased affinity with the south-central Andes and Titicaca basin region.

In all these analyses above, the UC-008 and UC-012 groups appeared genetically very similar. To further test if the individuals from UC-008 and UC-012 are genetically homogenous with respect to all other groups, we used the full 1,240k dataset and performed statistics of the form *f*_*4*_(*Mbuti, Test; UC12, UC8*) (Dataset S2*A*) and qpWave analyses (Dataset S2*B*) using the same strategy as for the individual-level grouping. These showed that UC8 and UC12 are a clade with each other and homogenous in ancestry, so we performed later analyses with them grouped together.

The prior analyses suggested that the sampled individuals from the Chincha Valley are of north or central coast ancestry, so we performed additional analyses to distinguish between these two. We calculated statistics of the type *f*_*4*_(*Mbuti, Test; Coast, Chincha*), where *Test* is any modern or ancient Native American or global population in both the 1,240k and Human Origins datasets, and *Coast* is *NorthPeruCoast*, *CentralPeruCoast*, or *SouthPeruCoast* as defined in Nakatsuka et al. (53). We found significant statistics (Dataset S3*A*) for many *Test* groups when “*Coast*” was *SouthPeruCoast* or *CentralCoast*, but not for *NorthPeruCoast*, meaning that *NorthPeruCoast* is consistent with being a clade with Chincha while the other ancient Peruvian coastal groups are not. This supports the notion that the sampled individuals are of Peruvian north coast ancestry.

We then performed qpWave analyses with the Chincha group paired with one of the groups defined in Nakatsuka et al. (53)—*NorthPeruCoast, NorthPeruHighlands, CentralPeruCoast, SouthPeruCoast, SouthPeruHighlands*)—with the other groups used as outgroups in addition to *Peru_Cuncaicha_4200BP, Peru_Lauricocha_5800BP, Karitiana, Peru_SanSebastian_450BP* (individuals from Cusco), and *Peru_Riollave_1700BP* (individuals from the Titicaca Basin) (49, 50, 52). In these analyses, rank 0 was rejected (*P* < 0.0005) for all pairs except *NorthPeruCoast* (*P* = 0.05) (Dataset S3*B*). In addition, we used qpAdm and attempted to model Chincha as a mixture of *NorthPeruCoast* and *CentralPeruCoast*. We found that Chincha was 1.025 ± 0.316 *NorthPeruCoast* and −0.025 ± 0.316 *CentralPeruCoast* ancestry (Dataset S3*C*). These analyses indicate that the Chincha individuals are most similar to ancient individuals of the Peruvian north coast. Since the Chincha individuals can be well modeled with only *NorthPeruCoast* ancestry, this means that they are unadmixed to the limits of our resolution. They lack a significant amount of additional ancestry from other regions, including the Peruvian south coast, which is the geographical region where they were found. This is further demonstrated by the lack of significantly negative statistics of the form *f*_*3*_(*Chincha; PeruRegion1, PeruRegion2*), which would normally arise if the Chincha individuals were a mixture of ancestry related to two different Peruvian regions (Dataset S3*D*).

Lastly, we found via *f*_*4*_-statistics and qpWave analyses (Datasets S3*A* and S3*B*) of the same form as above that the sampled individuals form a clade with the Torontoy1 individuals from Cusco and the Aconcagua individual (a sacrificial victim found in Argentina) (57), which were all Late Horizon individuals of Peruvian north coast ancestry associated with the Inca Empire (53).

Overall, our results indicate that the sampled individuals from the middle Chincha Valley are genetically homogeneous to the limits of our resolution and most genetically similar to ancient individuals of the north Peruvian coast. This is the same ancestry type found in a Late Horizon sacrificial boy found in Argentina (57) and two individuals from the Inca Period site of Torontoy in the Sacred Valley (53). As a limitation to our study, we note that ancient north coast individuals used for comparison were from the archaeological site of El Brujo and that ancient central coast individuals were from Lima. Our results demonstrate that the tested individuals are most similar genetically to ancient El Brujo individuals, but it could be that there were groups to the south of these (i.e., in between El Brujo and Lima) that are even more genetically similar to the Chincha individuals and thus better potential sources for their origin.

## Discussion

We employed a transdisciplinary, local-scale (individual-by-individual) approach that synthesizes multiple independent lines of evidence—ceramic, textile, strontium isotope, and genome-wide data alongside Colonial-era records—to investigate mobility in the Chincha Valley. These data are consistent in their support for the following hypothesis: nonlocal groups moved to the Chincha Valley prior to the Colonial period. Where did the six individuals from UC-008 Tomb 1 and UC-012 Tomb 33 come from? The strontium isotope data suggest they are from a coastal Andean region, while the more precise genome-wide analyses indicate that they likely originated from the north coast. Analyses of north coast-style ceramics and textiles documented in the Chincha Valley are consistent with these findings.

How did they get to the Chincha Valley? In addition to pedestrian travel, potential modes of transportation include llama caravans and oceangoing craft that probably hugged the coastline. Two caravanserais that may have served as loci for trade were excavated in the Chimú capital of Chan Chan (58). Members of the Chimú polity, perhaps merchants serving as retainers for lords (59), used balsa rafts potentially to acquire *Spondylus* (*Spondylus princeps*) shells from Ecuador and transport them across the Andes. The utility of this type of watercraft for the movement of goods (and thus people) has been called into question by Hocquenghem (60), who argues that they were more likely used by fisherfolk. Nevertheless, we suggest that the sampled individuals, if they came from the north coast, could have traveled to the Chincha Valley on foot and/or with oceangoing craft. These modes of transportation are not mutually exclusive.

Why did these individuals move to the Chincha Valley? Our data best fit a model of Inca resettlement. Written sources claim that *mitmaqkuna* were present in the Chincha Valley (16), and that the Inca moved north coast groups across the south coast (38–40). As an example of a *chaupiyunga* (an ecological area situated between coastal plains and highland valleys), the middle Chincha Valley has rivers and copper mines that would have made it a prime destination for the resettlement of northern water management specialists and miners. If this were the case, we would expect to encounter north coast-style textiles in archaeological contexts dating to the Late Horizon because the Inca reportedly required *mitmaqkuna* to wear traditional clothing (27). Indeed, several Chimú-style textiles from Late Horizon sites have been documented in the lower and middle Chincha Valley. Such results coincide with ceramic, strontium, and our genome-wide data from Chincha. We cannot genetically rule out that the tested individuals came from a group that voluntarily traveled to the study area prior to the Late Horizon. It remains plausible that traders from the north coast arrived in Chincha before the Inca. Our results, however, show that the individuals were of unadmixed (to the limits of our resolution) north Peruvian coast ancestry, providing greater support for a more recent immigration which is consistent with the historical and archaeological record. Our data do not appear to reflect “locals,” and future studies analyzing ancient individuals from the LIP and older time periods from the Chincha Valley would evaluate whether the ancestry of the groups that existed in Chincha prior to the Late Horizon was more characteristic of other south Peruvian coast groups. This study shows clear evidence of long-distance migration of the tested individuals, and future studies with larger sample sizes will allow us to determine how widespread this phenomenon was in Chincha, including whether population heterogeneity existed in the area.

More broadly, this paper contributes to the aim of integrating aDNA with more traditional archaeological and historical research by promoting—and demonstrating—a transdisciplinary, local-scale approach to studying mobility. All lines of evidence suffer from limitations that produce interpretive difficulties. Genomic, archaeological, biogeochemical, and historical data cannot individually explain ancient mobility in complex societies. Our transdisciplinary work represents an ideal case study that sets a methodological standard for examining ancient mobility because it synthesizes multiple data types and evaluates their consistency in support of models of movement. This study also raises additional questions: How many nonlocals moved to the Chincha Valley and over what time period? What percentage of the Chincha Valley population did they compose? How diverse were the communities that lived in this region? How did the arrival of nonlocals impact local social life? We recognize that this ideal approach cannot be used for all regions and time periods, but it remains crucial for producing new and diverse questions for future research that can in turn pave the way for deeper understandings of the past.

## Methods

### Samples.

The six samples analyzed here genetically were all teeth belonging to six different individuals found in UC-008 Tomb 1 from the UC-008 cemetery and UC-012 Tomb 33 from the UC-012 cemetery in the middle Chincha Valley. Surgical gloves and masks were worn during extraction and bagging of the samples to prevent contamination.

### Radiocarbon Dating.

We obtained direct ^14^C AMS dates for four samples. The tooth samples were prepared and measured at University of California Irvine (UCIAMS) using methods described by Beverly et al. (61). All radiocarbon ages were calibrated using OxCal version 4.3 (62). Since all samples were found just under 20 km from the Pacific Coast, elevated δ^15^N values are likely indicative of marine dietary components for the individuals. Values of δ^15^N provide a roughly linear scale of the relative importance of marine dietary resources, with ∼11.5‰ indicating a wholly terrestrial diet and ∼22.0‰ indicating a predominately (∼90%) marine diet. Therefore, dates were calibrated using a mixture of SHCal13 (63) and the Marine13 calibration curves (64) based on estimates ranging from 20 to 40 (±10)% marine dietary component, depending on the δ^15^N values (Dataset S1). Marine reservoir correction was performed using a ΔR value of 110 ± 49, derived from the most proximate marine reservoir study conducted in Paracas, Peru (65).

We recognize that other factors may have contributed to elevated δ^15^N values, such as aridity and fertilizer use (66). Guano fertilizer from the Chincha islands was a potential source of wealth (67), and could have been valuable to farmers in an environment like the Chincha Valley where arid coastal desert was made fertile through irrigation. However, the received values of δ^13^C and δ^15^N are consistent with a population subsisting primarily on terrestrial agriculture, supplemented in some degree by marine sources, and do not present any anomalous findings for a coastal population. We report dates calibrated using both the aforementioned mixing protocol (SHCal13 and Marine 13) as well as using only the SHCal13 curve (Dataset S1). Both sets of data are consistent with ascription to the Late Horizon.

### Ancient DNA Laboratory Work.

All samples were processed in the dedicated clean rooms at University of California Santa Cruz Paleogenomics in Santa Cruz (UC-PL), following strict procedures to minimize contamination (68). DNA was extracted from tooth roots using a method optimized to retain short DNA fragments (69), adding a 15-min predigestion step with 0.5% bleach solution as suggested by Boessenkool et al. (70). Partially uracil-DNA glycosylase (UDG) treated (43) dually indexed single-stranded DNA (ssDNA) libraries were built following the protocol by Troll et al. (71). The success of the library construction, quantity, and length was evaluated using a TapeStation 2200. The final pooled libraries were sequenced (2 × 150, paired end) on an Illumina HiSEq. 4000 at Fulgent Genetics (Temple City).

Base calling for the read data was done with Casava 1.8.2. We trimmed adapters and merged paired reads that overlapped by at least 15 nucleotides using SeqPrep (https://github.com/jstjohn/SeqPrep) taking the highest quality base to represent each nucleotide and then mapped the sequences to the human genome reference sequence (GRCh37 from the 1000 Genomes Project) using the same command of the Burrows–Wheeler aligner (BWA) (version 0.6.1) (72). We trimmed two nucleotides from the end of each sequence and randomly selected a single sequence at each site covered by at least one sequence in each individual to represent their genotype at that position (“pseudo-haploid” genotyping). All raw read processing was done employing the UC-PL inhouse computational pipeline (https://github.com/mjobin/batpipe).

### Data Authenticity.

Evidence for ancient DNA authenticity was evaluated by measuring the rate of damage in the first nucleotide, flagging individuals as potentially contaminated if they had a less than 3% cytosine-to-thymine substitution rate.

We estimated mtDNA contamination using contamMix version 1.0-12 (44). The software was run with down-sampling to 50× for samples above that coverage, −trimBases 2, 8 threads, 4 chains, and 2 copies, taking the first one that finished. For males we estimated X-chromosome contamination with ANGSD (45). We used the parameters minimum base quality = 20, minimum mapping quality = 30, bases to clip for damage = 2, and set all other parameters to the default.

### Comparative Data.

The genomes obtained for this study were merged with 1,240k genome-wide SNPs from 141 ancient, pre-Columbian individuals from 57 South and North American groups (49, 50, 53, 57, 73, 74). We further included present-day human data from the Simons Genome Diversity Project, which included 26 Native American individuals from 13 groups with high-coverage full-genome sequencing, and data from 224 Native American individuals from 34 different populations genotyped on the Affymetrix Human Origins array (54, 75, 76) for some analyses.

### Uniparental Markers.

Y-chromosome haplogroup calling and the identification of mitochondrial haplotypes followed the procedure described in Nakatsuka et al. (53). To determine the Y-chromosomal haplogroups, we determined the most derived mutation for each sample using the tree of the International Society of Genetic Genealogy (ISOGG) version 11.110 (accessed April 21, 2016) and confirmed the presence of upstream mutations consistent with the assigned Y-chromosome haplogroup. For the mitochondrial haplotypes we embedded the consensus mitochondrial genomes in the existing mitochondrial tree (mtDNA tree Build 17 [February 18, 2016]) using the online tool HaploGrep2 (77) to determine the haplotypes.

### ADMIXTURE Clustering Analysis.

We ran ADMIXTURE (78) for K = 2 to K = 10 with the merged HO dataset, after removing sites which did not have at least 70% of samples with a called genotype. We produced 100 replicates for each K value, reporting the replicate with the highest likelihood. Replications and automated filtering were performed using the UCSC-PL wrapper script adpipe.py (https://github.com/mjobin/UPA/blob/master/adpipe.py).

### Principal Component Analysis.

We performed PCA using the smartpca version 16680 in EIGENSOFT (79). We used the default parameters and the lsqproject: YES, and newshrink: YES options and performed PCA on the Human Origins dataset of present-day unadmixed Andean individuals (54). We projected the ancient individuals onto the principal components determined from the present-day individuals.

### Symmetry Statistics and Admixture Tests (*f*-Statistics).

We used the qp3pop and qpDstat packages in ADMIXTOOLS (80) to compute *f*_*3*_-statistics and *f*_*4*_-statistics (using the f4Mode: YES parameter in qpDstat) with SEs computed with a weighted block jackknife over 5-Mb blocks. Analyses were performed using the 1,240k SNP dataset. We used the inbreed: YES parameter to compute *f*_*3*_-statistics to account for our random allele choice at each position. We computed outgroup-*f*_*3*_ statistics of the form *f*_*3*_(*Mbuti; Pop1, Pop2*), to measure the shared genetic drift between population 1 and population 2. We created a matrix of the outgroup-*f*_*3*_ values between all pairs of populations and converted these to distances by taking the inverse of the values. We then generated a neighbor-joining tree using PHYLIP version 3.696’s (81) neighbor function and setting *USA_USR1_AncientBeringian_1140BP.SG* as the outgroup. In some of our analyses we plot the *f*-statistics on a heatmap using R (https://github.com/pontussk/point_heatmap/blob/master/heatmap_Pontus_colors.R).

### qpWave and qpAdm Analyses.

We used qpWave (56) from the ADMIXTOOLS package to test whether the individuals from the two Chincha contexts form a genetically homogenous group with each other, and if they form a homogenous group with any of the regional Andean groups: *NorthPeruCoast*, *NorthPeruHighlands*, *CentralPeruCoast*, and *SouthPeruCoast*, as defined in Nakatsuka et al. (53). For all qpWave analyses, we used the default settings except for the change that we set allsnps: YES. Different combinations of ancient Andean groups were used as outgroups. We used qpAdm (47) in the ADMIXTOOLS package to estimate the proportions of ancestry in a *Test* population deriving from a mixture of N “reference” populations by taking advantage of the fact that they have shared genetic drift with a set of outgroup populations. We set the details: YES parameter, which reports a normally distributed Z-score for the fit (estimated with a block jackknife).

### Data and Materials Availability.

All sequencing data are available from the European Nucleotide Archive, accession number PRJEB37726.

## Supplementary Material

Supplementary File

Supplementary File

Supplementary File

Supplementary File
