# Supplementary material for: Integration of ancient DNA with transdisciplinary dataset finds strong support for Inca resettlement in the south Peruvian coast
Source: Proc Natl Acad Sci U S A. 2020 Jul 13;117(31):18359–68. doi: 10.1073/pnas.2005965117 (PMC7414190; doi:10.1073/pnas.2005965117)
Supplement: Supplementary File [file pnas.2005965117.sapp.pdf]

Supplementary Information for

Integration of ancient DNA with transdisciplinary dataset finds strong support for Inca resettlement in the south Peruvian coast

Jacob L. Bongers, Nathan Nakatsuka, Colleen O'Shea, Thomas K. Harper, Henry Tantaleán, Charles Stanish, and Lars Fehren-Schmitz

Jacob L. Bongers
Email: J.Bongers@uea.ac.uk

This PDF file includes:

Figures S1 to S2

Other supplementary materials for this manuscript include the following:

Datasets S1 to S3

www.pnas.org/cgi/doi/10.1073/pnas.2005965117

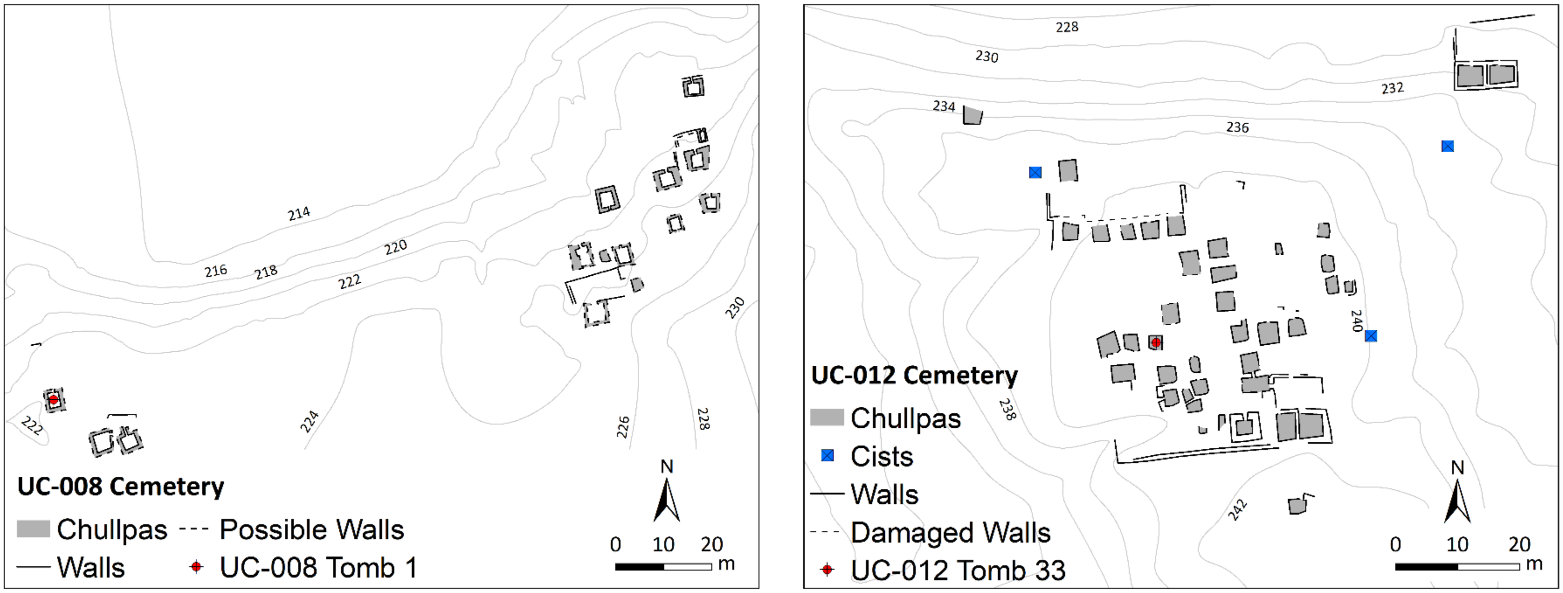

Figure S1. Site plans for the UC-008 and UC-012 cemeteries, showing the locations of UC-008 Tomb 1 and UC-012 Tomb 33.

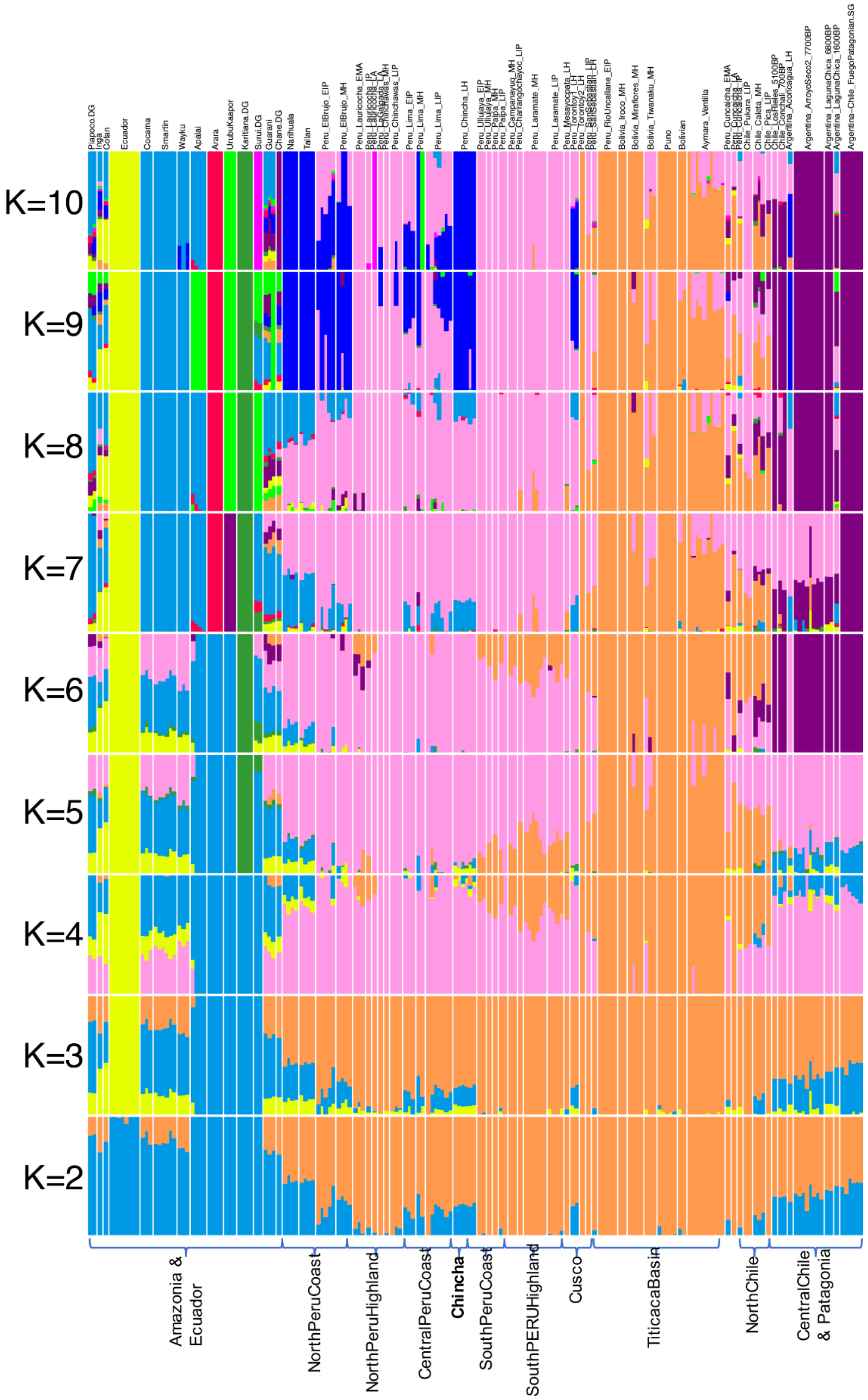

Figure S2. ADMIXTURE plot at different K values. The run with the highest log-likelihood score after 100 trials was taken for each K value.

Dataset S1 (separate file). Sample and sequencing details

Dataset S2 (separate file). qpWave homogeneity

Dataset S3 (separate file). qpWave and qpAdm characterization
